# Primary intercavernous lymphoma of the central nervous system

**DOI:** 10.1590/0100-3984.2014.0078

**Published:** 2015

**Authors:** Arthur Henrique de Aquino Dultra, Fabio Noro, Alessandro Severo Alves de Melo, José Alberto Landeiro, Edson Marchiori, Marilene Filgueira do Nascimento

**Affiliations:** 1 Hospital Copa D’Or, Rio de Janeiro, RJ, Brazil.; 2 Universidade Federal do Rio de Janeiro (UFRJ), Rede D’Or, Rio de Janeiro, RJ, Brazil.; 3 Universidade Federal Fluminense (UFF), Niterói, RJ, Hospital Barra D’Or, Rio de Janeiro, RJ, Brazil.; 4 Universidade Federal Fluminense (UFF), Niterói, RJ, Brazil.; 5 Instituto Nacional de Câncer (INCA), Rio de Janeiro, RJ, Brazil.

*Dear. Editor*,

A male, 63-year-old, HIV-negative patient was admitted to the hospital with intermittent frontal headache, right facial pain and diplopia for at least two months. Neurological examination revealed both cranial nerves VI paresis and facial pain in the area innervated by the branches V2 and V3 of the right trigeminal nerve. No alteration was observed in the other cranial nerves. The patients presented with normal gait and unaltered muscle strength and balance. Laboratory tests and cerebrospinal fluid puncture revealed normal results.

Cranial magnetic resonance imaging (MRI) demonstrated an expansile, intrasellar homogeneous, solid, well delimited lesion, with isosignal on T1- and T2-weighted sequences with a lower enhancement pattern in relation to the hypophysis. The lesion occupied both cavernous sinuses, particularly at right. The way in which the lesion extended suggested the involvement of the intercavernous sinus (Figure 1). The patient was submitted to surgical treatment, with an uneventful postoperative period. Anatomopathological analysis (Figure 2A) demonstrated diffuse non-Hodgkin's B-cell lymphoma. The patient underwent complementary radiotherapy, and successive follow-up with MRI did not demonstrate lesion recurrence in up to five years (Figure 2B).

**Figure 1:**
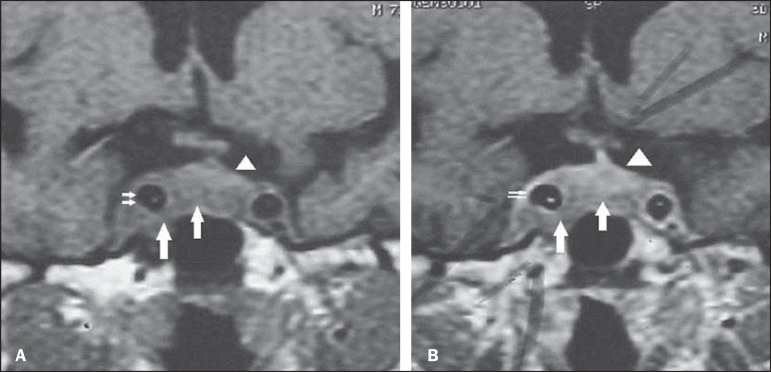
Pre-resection coronal magnetic resonance imaging T1-weighted sequence, before (**A**) and after (**B**) contrast agent injection: homogeneous, solid mass (large arrows) showing intermediate signal intensity in the right cavernous sinus, involving the right internal carotid artery (small arrows), with contralateral extension under the hypophysis. The gland (arrowheads) is superiorly displaced by the mass.

**Figure 2:**
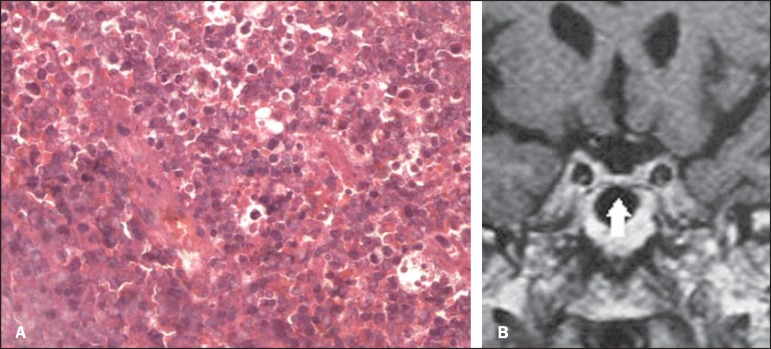
**A:** Histopathological analysis demonstrates the presence of cells with large nuclei, sometimes with centrally located nucleoli and basophilic cytoplasm. **B:** Six-month postoperative and post-radiotherapy follow-up. Coronal magnetic resonance imaging T1-weighted sequence: no sign of the lesion is seen neither in the sellar cavity nor in the cavernous sinuses. The hypophysis is slightly reduced in volume (arrow).

Central nervous system (CNS) lymphoma is a quite rare neoplasm, affecting most the supratentorial region. In the case of immunocompetent patients, CNS lymphoma manifests as a single, solid lesion, generally without necrosis, with hypo/isosignal on T2- and isosignal on T1-weighted sequences, and intense contrast-enhancement^(1,2)^.

Anatomically, cavernous sinuses are irregularly-shaped, trabeculated/compartmentalized venous sinuses located along the lateral aspect of the sella turcica^(3)^. Cranial nerves III, IV, V1 and V2 are located within the lateral dural wall, and not within the cavernous sinus^(3)^. The two cavernous sinuses communicate with each other via anterior and posterior intercavernous venous plexuses^(3)^. Such connections allow for extension of the inflammatory or neoplastic process to the contralateral sinus^(4)^.

In the case of cavernous sinus lymphoma, considering the proximity to several cranial pairs, cranial nerve lesions may be observed; however this is an uncommon finding^(1)^.

CNS lymphomas are rarely found, particularly in immunocompetent individuals. In the present case, the unprecedented characteristic is the infra-hypophyseal involvement by dissemination through the intercavernous sinus. Despite its rarity, particularly in immunocompetent individuals, the finding of a solid, homogeneous lesion with isosignal on T1- and T2-weighted sequences in the region of the cavernous sinuses should raise the hypothesis of lymphoma.

## References

[1] Reis F, Schwingel R, Nascimento FBP (2013). Central nervous system lymphoma: iconographic essay. Radiol Bras.

[2] Barreira Junior AK, Moura FC, Monteiro MLR (2011). Linfoma não-Hodgkin bilateral do seio cavernoso como
manifestação inicial da síndrome de imunodeficiência
adquirida: relato de caso. Arq Bras Oftalmol.

[3] Osborn AG (2014). Encéfalo de Osborn. Imagem, patologia e anatomia.

[4] Rocha AJ, Vedolin L, Mendonça RA (2012). Encéfalo.

